# Therapeutic Strategies for Diabetes: Immune Modulation in Pancreatic β Cells

**DOI:** 10.3389/fendo.2021.716692

**Published:** 2021-08-17

**Authors:** Sugyeong Jo, Sungsoon Fang

**Affiliations:** ^1^Department of Medical Science, Brain Korea 21 Project for Medical Science, Yonsei University College of Medicine, Seoul, South Korea; ^2^Severance Biomedical Science Institute, Gangnam Severance Hospital, Yonsei University College of Medicine, Seoul, South Korea

**Keywords:** diabetes, pancreatic β cell, immune modulation, autoimmunity, inflammation, gut microbiota

## Abstract

Increased incidence of type I and type II diabetes has been prevailed worldwide. Though the pathogenesis of molecular mechanisms remains still unclear, there are solid evidence that disturbed immune homeostasis leads to pancreatic β cell failure. Currently, autoimmunity and uncontrolled inflammatory signaling pathways have been considered the major factors in the pathogenesis of diabetes. Many components of immune system have been reported to implicate pancreatic β cell failure, including helper T cells, cytotoxic T cells, regulatory T cells and gut microbiota. Immune modulation of those components using small molecules and antibodies, and fecal microbiota transplantation are undergoing in many clinical trials for the treatment of type I and type II diabetes. In this review we will discuss the basis of molecular pathogenesis focusing on the disturbed immune homeostasis in type I and type II diabetes, leading to pancreatic β cell destruction. Finally, we will introduce current therapeutic strategies and clinical trials by modulation of immune system for the treatment of type I and type II diabetes patients.

## Introduction

Diabetes is a complex disorder caused by multiple genetic and environmental factors. For example, sex differences in genetic and environmental factors such as sex hormones, sex chromosomes and sex-specific epigenetic modification are associated with the development of diabetes (1–4). Although there are numerous risk factors for diabetes, dysfunction of pancreatic β cells is a common feature of both type 1 diabetes (T1D) and type 2 diabetes (T2D). Pancreatic β cells play crucial roles in the regulation of glucose homeostasis. They have molecular sensors to recognize a rise in blood glucose and to produce insulin for the maintenance of blood glucose levels. While destruction of pancreatic β cells leads to development of T1D, T2D occurs when β cells fail to secrete sufficient insulin to compensate for insulin resistance. Numerous studies have shown that multiple molecular mechanisms, including autoimmune, inflammation and metabolic stress are risk factors for the development of β cell failure. Autoimmune-mediated β cell dysfunction is due to β cell autoantigens and immune cell infiltration of the pancreatic islets (insulitis) (5). The autoantibodies have been identified in the sera of T1D patients and these autoantibodies serve as biomarkers for the prediction of T1D (6). Besides, a spontaneous animal model of T1D, non-obese diabetic (NOD) mice has shown the presence of autoreactive T cells and antigen-presenting cells (APCs) in islets, implying that autoreactive T cells recognize β cell autoantigen to infiltrate islets and contribute to β cell destruction for the development of T1D (7). In addition to T1D, islet inflammation also contributes to β cell dysfunction in T2D. Infiltration of macrophages and increased expression of pro-inflammatory cytokines have been observed in islets of rodent models including high-fat diet (HFD)-fed mice, db/db mice and GK rat (8, 9). Glucotoxicity and lipotoxicity have been shown to induce β cell death in T2D. Chronic exposure of β cells to high glucose altered glucose-stimulated insulin secretion (GSIS) (10). Although short-term exposure to saturated free fatty acids (FFAs) is able to stimulate insulin secretion, prolonged exposure to saturated FFAs dramatically suppresses GSIS by increasing oxidative stress in β cells (11).

Recent studies have reported that the gut microbiota is associated with both T1D and T2D (12–14). The microbiota plays a critical role to regulate energy metabolism by fermentation of carbohydrates and production of metabolites such as bile acids, short-chain fatty acids (SCFAs) including acetate, propionate, and butyrate. The metabolites induced by gut microbiota are involved in glucose homeostasis. For example, SCFAs stimulate secretion of glucagon-like peptide 1 (GLP-1), an incretin to potentiate insulin secretion for glucose homeostasis (15). Thus, alteration of gut microbiota may contribute to disturbed incretin-mediated insulin signaling in diabetes patients (16–18). And, it has been reported that children with high genetic risks for developing T1D have distinct features of microbiome compared with a healthy control group (19). Though the underlying molecular mechanisms are still unclear, the alteration of gut microbiota in early life has been shown to correlate with the presence of autoantibodies and autoimmunity in pancreatic islets, suggesting that gut microbiota plays a pivotal role to modulate immune responses in islets for the development of T1D (20). Likewise, numerous studies using metagenomics and 16S rRNA-based high-throughput sequencing have reported compositional and functional changes of gut microbiota in T2D patients (17, 21, 22). Cohort studies from Europe and China commonly demonstrated a decrease of butyrate-producing gut bacteria in T2D patients compared to the normal group (21–23). The reduction of butyrate-producing gut bacteria contributed to both T1D and T2D development, implying that gut microbiota alteration is crucial risk factor for the pathogenesis of diabetes.

Herein, we will provide an overview of the pathogenesis and therapeutic strategies of diabetes in terms of immune modulation using gut microbiota. Given that immune modulation strongly contributes to the destruction of pancreatic β cells for the development of diabetes, the crosstalk between the gut microbiota and immune system would be promising therapeutic strategy for the treatment of diabetes.

## The Pathogenesis of Diabetes

### Autoimmunity in Type I Diabetes

It has been widely accepted that T1D is an organ-specific autoimmune disease. Autoantibodies in pancreatic islet serve as diagnosis markers to predict the pathogenesis of T1D in both NOD mice and T1D human patients (24, 25). These autoantibodies recognize specific self-antigens include insulin, glutamic acid decarboxylase (GAD), zinc transporter (ZnT8), and insulinoma-antigen 2 (IA-2) in pancreatic islets. Though the molecular mechanisms of how autoantigens are processed remain still unclear, endoplasmic reticulum (ER) stress-mediated misfolded proteins in pancreatic β cells may contribute to the process of these self-antigens (26–28). Antigen-presenting cells (APCs) such as macrophages and dendritic cells (DCs) have been shown to infiltrate in β cells and present autoantigens to naïve CD4^+^ T cell for T cell activation. After recognizing autoantigens, naïve CD4^+^ T cell differentiate into autoreactive CD4^+^ T cells, leading to promote B cells to produce autoantibodies and activate CD8^+^ T cells to differentiate into cytotoxic T cell (5). These autoreactive CD4^+^ T cells and CD8^+^ T cells are key drivers of autoimmune reaction to destroy pancreatic β cells.

Recently, it has been reported that polymorphism of Human leukocyte antigens (HLA) class II encoding DQ and DR is a genetic determinant of T1D (29). In NOD mice, a peptide derived from insulin was reported to serve as one of the autoantigens. This peptide was able to bind major histocompatibility complex (MHC) class II molecule H2-A^g7^, resulting in activation of CD4^+^ T cells (30). HLA DR4-restricted CD4^+^ T cells from T1D patients are also able to respond to pre-proinsulin (PPI)-derived epitope (31). Once activated by self-antigens, the activated CD4^+^ T cells then secrete interleukin-2 (IL-2) to provide ‘help’ CD8^+^ T cell activation.

In addition to CD4^+^ T cells, cytotoxic CD8^+^ T cells are predominant lymphocytes infiltrating the pancreatic islets, resulting in β cell destruction. HLA class I has been shown to be overexpressed in pancreatic islets of early T1D patients, resulting in infiltration of CD8^+^ T cells and insulitis (32). Thus, MHC class I-deficient NOD. β2M^−/−^ mice decreased infiltration of CD8^+^ T cells into pancreatic islets, leading to ameliorate pathogenesis of T1D (33).

Recently, it has been reported that β cell peptides including insulin B chain, GAD and Islet-specific glucose-6-phosphatase catalytic subunit–related protein (IGRP) are prone to bind HLA-A2 in T1D patients and autoreactive CD8^+^ T cell response to many HLA-A*0201–restricted β cell peptides (34). Once activated, autoreactive CD8^+^ T cells release cytotoxic granules such as perforin, granzyme B and proinflammatory cytokines, including IFN-γ leading to destruction of pancreatic β cells (35). These proinflammatory cytokines increase the expression levels of MHC class I and chemokine CXCL10 which promote T cell infiltration in human β cells (32).

On the other hand, FOXP3^+^ regulatory T cells (Tregs) are known as immunosuppressive cells. Treg directly suppress the proliferation and activation of effector T cells or dendritic cells and secrete anti-inflammatory cytokines such as IL-10 (36). However, the function of FOXP3^+^ Treg is altered in the onset of T1D and the dysfunction of Treg may contribute to the pathogenesis of T1D (37, 38).

### Inflammatory Signaling in Type II Diabetes

Type 2 diabetes (T2D) is a multifactorial disease that resulted from the combination of both genetic and environmental factors. This disease is associated with hyperglycemia and is characterized by insulin resistance and dysfunction or death of β-cells, leading to insufficient insulin secretion for glycemic homeostasis.

Insulin resistance is highly associated with diet-induced obesity (39, 40). In the condition of obesity, dysfunction of adipose tissue which is characterized by enlarged adipocytes and abnormal secretion of adipokines and inflammatory cytokines promotes the progression of insulin resistance (41, 42). Adipocyte hypertrophy generally increase the expression of pro-inflammatory cytokines including tumor-necrosis factor-α (TNF-α), IL-6, and IL-1β in the human patients with insulin resistance. Furthermore, the adipocytes release macrophage chemoattractant protein-1 (MCP-1) to promote macrophage infiltration in adipose tissue (43). The infiltration of macrophages with secretion of proinflammatory cytokines contribute to insulin resistance, leading to increase of lipolysis and plasma FFA in the T2D patients (44).

Similar with adipose tissues, dysfunction of pancreatic β cells in T2D is involved in islet inflammation through increased proinflammatory cytokines and infiltration of immune cells. The islet inflammation has been observed in several T2D mouse model including *Psammomys obesus* rat, HFD-fed mice, db/db mouse and GK rat (45). Chronic hyperglycemia induces the production of IL-1β in pancreatic β cells which is implicated in insulin resistance as well as β cell dysfunction (46). Treatment of IL-1 receptor antagonist to HFD-fed mice resulted in protection from apoptotic β cell death and improved GSIS secretion by blocking IL-1β signaling (47). IL-1 receptor antagonisms inhibited macrophage infiltration into β cell, leading to improved hyperglycemia in GK rats (48). In addition, exposure of isolated human islets to high glucose levels resulted in an increase of IL-1β secretion to activate NF-κB, a key regulator of inflammation, and Fas (CD95) signaling to induce dysfunctional β cell (49). These findings suggest that glucotoxicity implicates an islet inflammatory process in T2D.

**Figure 1:**
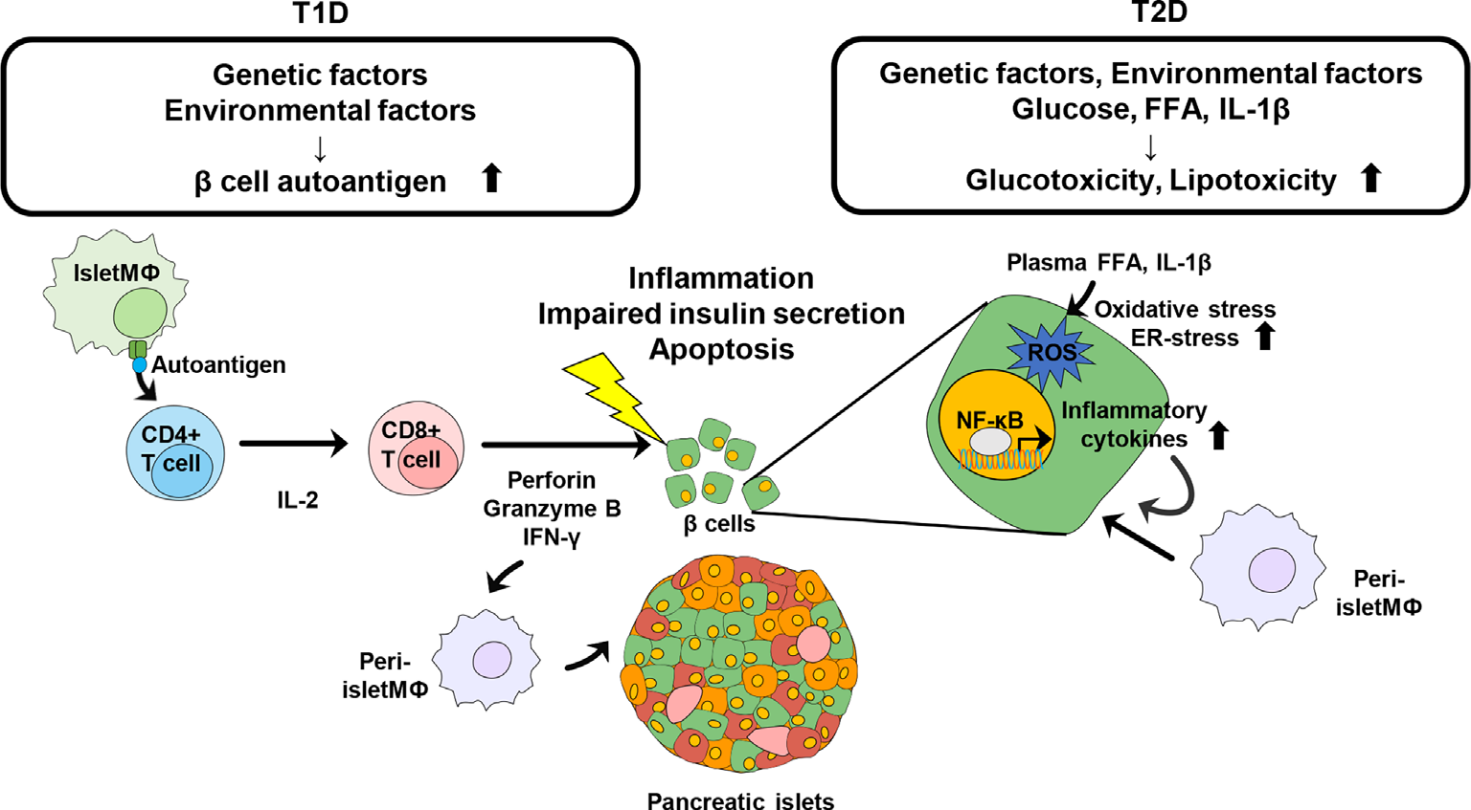
Schematic view of Immune modulation contributing to the destruction of pancreatic β cells in diabetes. Genetic and environmental factors contribute to generation of islet autoantigens. Intra islet macrophage or DC recognize the autoantigen and present it to naïve CD4+ T cells. Activated CD4+ T cells further activate CD8+ T cells to directly damage β cells and further induce the infiltration of other immune cells, leading to progression of T1D. Obesity is a risk factor of pathogenesis of T2D. Increased glucose, plasma FFA, and IL-1β promote oxidative stress and ER-stress in pancreatic β cells to induce insulin resistance as well as β cell destruction. Inflammatory cytokines recruit other immune cells into pancreatic islets and β cells and trigger further inflammation.

Likewise, lipotoxicity also has physiological impacts on the function of β cells. FFAs as well as TAG and cholesterol levels have been shown to be elevated in HFD-fed mice (47). Increase of plasma FFAs contributes to insulin resistance and impaired insulin secretion by inducing ER stress and oxidative stress. Moreover, excessive level of FFAs in skeletal muscle serves as toxic lipids such as ceramide and diacylglyceride, leading to incomplete fatty acid oxidation (50). These toxic FFA metabolites and incomplete fatty acid oxidation contribute to ER stress, oxidative stress and the generation of reactive oxygen species (ROS). Metabolic products of FFAs activate pro-inflammatory signaling pathways such as PKC and JNK leading to impaired insulin signaling. Saturated FFA also can activate toll-like receptor (TLR) signaling in β cells, followed by immune responses (51). FFA-induced TLR-mediated signaling has been shown to promote infiltration of macrophages through the secretion of chemokines such as CCL2 and CXCL1 in β cells (51). TLR4-mediated signaling also induced inflammation through increased production of pro-inflammatory cytokines such as IL-1β and IL-6. Thus, insulin resistance-mediated hyperglycemia and lipotoxicity induce inflammatory signaling to disturb pancreatic β cell homeostasis, leading to β cell failure in T2D patients (**Figure 1**).

### Immune Modulation by Microbiota

Recently, several studies have reported compositional changes of gut microbiota in both T1D and T2D and the alteration may contribute to the development of diabetes (52–54). The gut microbiota interacts with the host immune system *via* multiple mechanisms involved in TLR-mediated signaling and microbial products such as SCFAs (55, 56). In a rodent study, knock out of Myd88, an adaptor protein for multiple TLRs, has been shown to protect NOD mice from the development of T1D in specific pathogen free (SPF) condition. However, these Myd88-deficient NOD mice (NOD.Myd88^−/−^) in germ free condition developed T1D, implying that gut microbiota plays a pivotal role in pathogenesis of T1D in NOD mice (57). These findings propose that Myd88-dependent TLR signaling is crucial to T1D development and microbiota is required for the protective effects in the absence of TLR signaling in Myd88-deficient NOD mice.

Gut microbiota-mediated TLR signaling pathways also regulate the development of T2D. Lipopolysaccharides (LPS), a component of Gram-negative bacteria are known to promote inflammation by induction of pro-inflammatory cytokines (58). In a rodent study, it has been observed that HFD can change the composition of gut microbiota and increase plasma LPS concentration, resulting in low grade chronic inflammation (59). Microbiota-induced LPS binds to CD14/TLR4 complex and activates pro-inflammatory pathways leading to insulin resistance and β cell dysfunction (60, 61). In addition, HFD feeding reduced the expression of genes related to intestinal tight junction proteins and increases intestinal permeability, leading to impaired gut epithelial barrier (62). This impaired gut barrier also increases plasma LPS levels leading to LPS-induced inflammation and insulin resistance (63, 64). Thus, the gut microbiota-mediated LPS signaling pathways can modulate pro- or anti-diabetogenic pathways through multiple inflammatory signaling pathways.

The gut microbiota produces SCFAs by fermentation of nondigestible carbohydrates. The SCFAs including acetate, propionate and butyrate have an influence on immune systems in both T1D and T2D. Acetate can prevent T1D by reducing the population of autoreactive T cells in the pancreatic lymph node (65). The concentration of IL-21, an inflammatory cytokine contributing to T1D pathogenesis was also reduced in acetate-fed NOD mice. In addition to acetate, butyrate can also increase IL-10RA expression and potentiate integrity of intestinal epithelial cell (IEC) barrier through IL-10 signaling, leading to reduction of gut permeability (12, 66). Butyrate has also been reported to promote the generation of regulatory T cells which may be involved in autoimmunity suppression in T1D and lactate can be converted to butyrate by intestinal microbiota (67, 68). Thus, a low abundance of lactate- and butyrate-producing bacteria was observed within the gut microbiota of children with islet autoantibodies (20). As consistent, rodent study reported that the concentration of acetate and butyrate in germ free NOD.Myd88g^−/−^ is much lower than in SPF ones (65). Altogether, these results clearly proposed that microbiota modulates autoimmunity through metabolites, such as acetate and butyrate in T1D.

SCFAs also play pivotal roles during pathogenesis of T2D. SCFA binds to FFAR2 or FFAR3, G protein-coupled receptors which are expressed in gut, pancreas, white adipose tissue, and immune cells (69). SCFA-mediated FFAR2 and/or FFAR3 signaling stimulate the secretion of glucagon-like peptide-1 (GLP-1), a well-known incretin from the intestinal L cells, to potentiate insulin secretion from pancreatic β cells. Thus, SCFA-mediated GLP-1 secretion leads to decrease the incidence of T2D by ameliorating glucose homeostasis. In addition to SCFAs, the secretion of GLP-1 can be stimulated by other gut microbial metabolic products, such as hydrogen sulfide(H_2_S), a gas metabolite (70).

**Figure 2:**
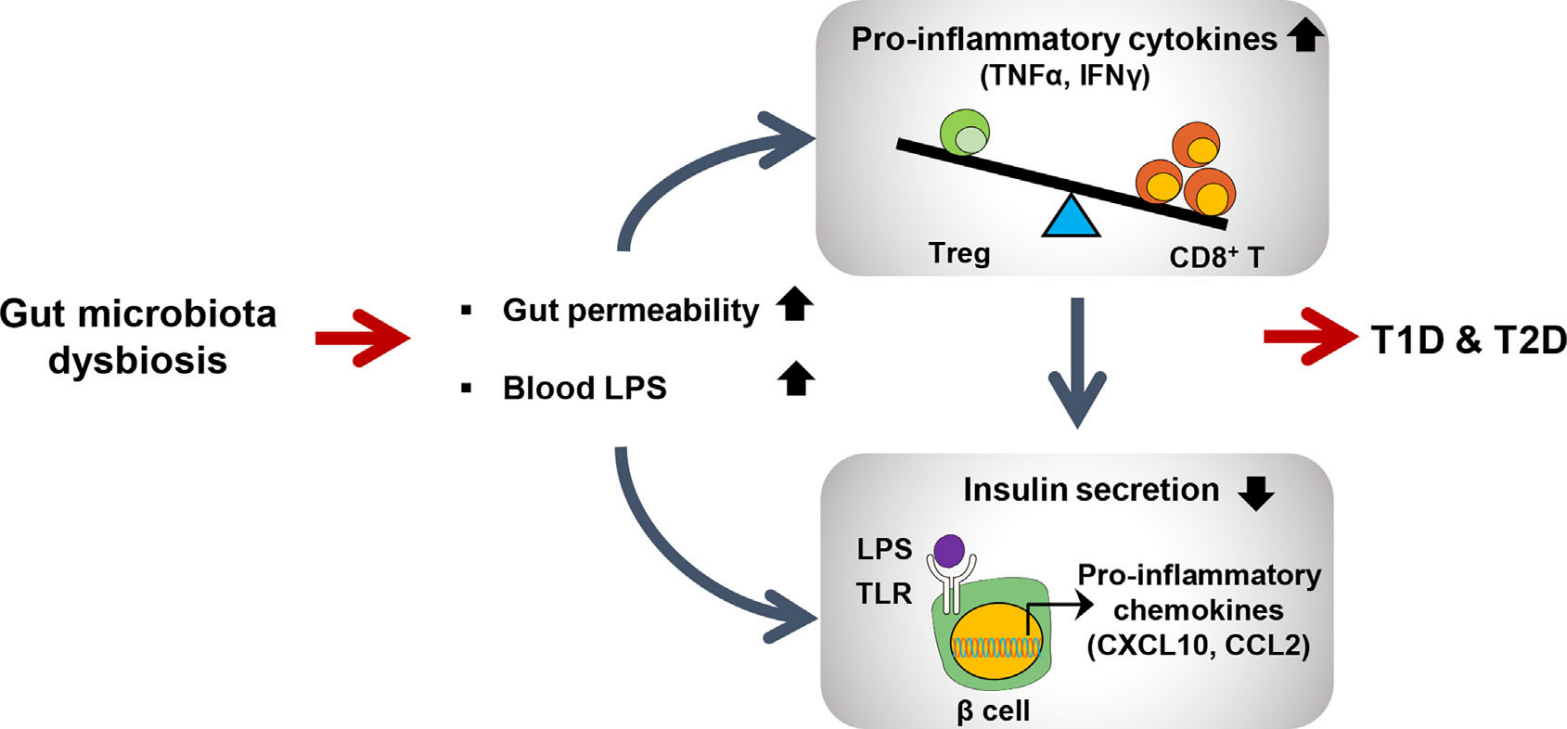
Schematic view of gut microbiota dysbiosis contributing to both type 1 and type2 diabetes. Dysbiosis leads to increased intestinal permeability and LPS level in blood. Elevated LPS level activates CD8+ T cells to produce proinflammatory cytokines to further activate immune signaling pathways in many peripheral tissues including pancreatic beta cells. LPS-induced TLR signaling in pancreatic beta cells promotes inflammation to reduce insulin secretion.

Gut microbiota regulates host bile acid metabolism through farnesoid x receptor (FXR) and TGR5 receptor. In general, primary bile acids are synthesized in the liver from cholesterol by CYP7A1, rate-limiting enzyme in the bile acid biosynthetic pathway, followed by conjugation to glycine and taurine for the secretion into intestinal lumen upon food intake (71). In the intestinal tract, gut microbiota converts primary bile acids to secondary bile acids. While primary bile acids prefer to bind FXR suppresses the hepatic synthesis of bile acids, secondary bile acids tend to bind TGR5 receptor and stimulates GLP-1 secretion from intestinal L-cells (72) (**Figure 2**).

## Therapeutic Strategies Using Immune Modulation in T1D

### Anti-CD3 Antibody

CD4^+^ T and CD8^+^ effector T cells are key factors of pancreatic β cell destruction and overt T1D development. Therefore, targeting T cells has been considered the most effective approach to the progression of T1D. Anti-CD3 monoclonal antibody (mAb) is a one of promising therapeutic approaches targeting T cells. Although the exact mechanism of anti-CD3 mAb is not clear, it has been considered to deplete autoreactive T cells and preserve regulatory T cell activity (73). In NOD mouse, treatment of anti-CD3 mAb induced long-lasting remission to ameliorate diabetic pathogenesis including restoration of normoglycemia (74). Thus, numerous human clinical studies using humanized anti-CD3 mAb has been reported for the treatment of pancreatic β cell destruction (74). In 1986, muromonab-CD3(Orthoclone OKT3), the first antibody recognizing CD3 molecules on human T cells, was approved in the US, but it is currently not available cause its side effects such as cytokine storm. Therefore, two humanized anti-CD3 antibodies engineered to improve the side effect of OKT3 called teplizumab and otelixizumab were developed. These antibodies retained binding regions of OKT3 have been investigated in numerous clinical studies for the prevention of T1D and resulted in preserved β cells (75–78).

### Anti-CD20 Antibody

B cells act as APC and have a pathogenic role in T1D. Therefore, approaches targeting B cells also have been taken to alter T1D progression. Anti-CD20 or anti-CD22 therapy showed prevention or reversal of T1D in NOD by depleting B (79, 80). Especially, treatment of rituximab, anti-CD20 mAb, transiently depleted B cells and delayed the rate of C peptide decline in T1D compared with a placebo group, although these effects were diminished after 2 years (81).

### Anti-Thymocyte Globulin (ATG)

Anti-thymocyte globulin (ATG) is another approach to target T cells for the prevention of pancreatic β cell destruction. ATG is rabbit-derived IgG against human T cells and thymocytes. ATG has been reported to deplete T cells through apoptosis and expand regulatory T cells (79, 82). Indeed, in a clinical trial, low-dose ATG has been shown to reduce glycated hemoglobin (Hb1Ac), a clinical marker of glycemic control, up to 2 years compared to placebo (80).

### Fusion Proteins: Alefacept and Abatacept

Fusion proteins, alefacept and abatacept also can deplete T cells and preserve β cell function. Alefacept consists of LFA-3 molecules and Fc portion of IgG and binds CD2 of effector memory T cell (83). Administration of alefacept led to increase of Treg/Teff ratio compared with placebo treatment (84). Abatacept is a CTLA4-IgG fusion protein that inhibits APC function to prevent T cell activation by blocking CD80 and CD86 on APC (85). In a clinical trial, the group of abatacept maintained lower HbA1c level compared with placebo after 3 years from T1D diagnosis (86). In addition, decreased level of follicular helper T cells involved in proliferation and isotype switching of B cells, was found in abatacept-treated T1d patients (87). These findings suggest that targeting T cells can modulate autoimmunity to prevent pancreatic β cell failure, although most of the above clinical trials failed to reach the endpoints in T1D.

### Autoantigen-Specific Therapies

β cell autoantigen-specific therapies is promising strategies to suppress autoimmune responses by selectively suppressing autoreactive T cell activation or expanding Treg (88, 89). One of these approaches has been applied with a peptide vaccine. For example, vaccination with insulin mimotope, an antigen-mimicking peptide, resulted in the prevention of T1D in NOD by converting naïve T cells into FOXP3^+^ Treg (90). Other novel insulin mimotopes also promoted induction of insulin-specific FOXP3^+^ Treg in humanized mice (91).

Furthermore, APC-based therapies with DCs and immunosuppressive macrophages and have attracted clinical interests in T1D. Administration of tolerogenic DC loaded with encoding autoantigen can suppress autoreactive T cells. T1D patients treated tolerogenic DCs which can induce proinsulin-specific Treg maintained C peptide level for 6 months (92, 93). Adaptive transfer of immunosuppressive macrophages prevented T1D in NOD through anti-inflammatory responses (94).

Administration of cytokines such as IL-2 also prevents autoimmunity in T1D. IL-2 is essential not only to the activation of T cells but also the expansion of FOXP3^+^ Treg. It has been reported that FOXP3^+^ Treg preferentially responds to low-dose of IL-2 versus T cells (95). In NOD studies, low-dose of IL-2 increased Treg population to prevent the progression of diabetes (95). Furthermore, administration of low dose of aldesleukin, a recombinant IL-2, increased the population of Treg without drug-relative adverse effects in T1D patients versus placebo (96). Currently, a phase 2 clinical trial evaluating the effect of ultra-low dose aldesleukin on the preservation of β cell function is ongoing (NCT03782636) (96) (**Table 1**).

**Table 1:** Summary of major clinical trials for anti-inflammatory therapies on T1D.

Agent	Mechanism of Action	Phase, ID	Main Findings	Reference
Teplizumab	Anti-CD3 mAb	Phase III, NCT00385697	Slowed reduction of C peptide	(97)
		Phase II, NCT00129259	Slowed reduction of C peptide	(98)
		Phase II, NCT01030861	Delayed progression	(76)
		Phase III, NCT03875729	Ongoing	(99)
Otelixizumab	Anti-CD3 mAb	Phase III, NCT00678886	Failed to meet primary end point	(77)
		Phase III, NCT01123083	Failed to meet primary end point	(100)
Alefacept	Anti-CD2 fusion protein	Phase II, NCT00965458	Slowed reduction of C peptide	(101)
Abatacept	CTLA4-Ig	Phase II, NCT00505375	Slowed reduction of C peptide	(102)
Rituximab	Anti-CD20 mAb	Phase II, NCT00279305	Slowed reduction of C peptide	(81)
		Phase II, NCT03929601	Delay	(103)
Aldesleukin	Low-dose of IL-2	Phase I/II, NCT01827735	Dose-dependently enhanced Treg function	(104)

mAb, monoclonal antibody.

## Anti-Inflammatory Therapeutic Strategies for T2D

T2D is a chronic metabolic disorder associated with obesity. Abnormal fat accumulation in adipose tissue and non-adipose organs including liver and pancreatic β cells promotes inflammatory responses such as the production of pro-inflammatory cytokines and chemokines, resulting in insulin resistance and β cell dysfunction. Therefore, anti-inflammatory approaches have been considered as a therapeutic strategy for the prevention of T2D.

### Metformin

Metformin is one of the most common drugs for the treatment of T2D. Metformin reduces blood glucose levels by suppressing hepatic glucose production and improving insulin sensitivity. In addition, metformin has a pivotal role in anti-inflammatory responses through inhibition of NF-κB signaling (105). Although the mechanisms of metformin remain unclear, it has been widely accepted that metformin acts *via* activation of AMP-activated protein kinase (AMPK). AMPK plays a potent anti-inflammatory effect by inhibiting NF-κB and regulating redox balance (106). Treatment of metformin increases phosphorylation of AMPK, enhances insulin sensitivity, and decreased ceramide and DAG related to saturated FFA in HFD-fed mice (107). Another study also demonstrated that metformin treatment increased Treg population and decreased Th17 by inducing FGF21 production in HFD-fed mice (108).

### GLP-1 Receptor Agonist & DPP4i

GLP-1 receptor agonists (GLP-1 RAs) and dipeptidyl peptidase 4 inhibitors (DPP-4is) have been widely prescribed for the T2D patients in clinic. GLP-1 RA such as liraglutide and exendin-4 binds to GLP-1 receptor in pancreatic β cell to stimulate Ca^2+^ influx and insulin secretion (109). Furthermore, GLP-1 RA has anti-inflammatory effects by suppressing expression of proinflammatory cytokines and chemokines. Exendin-4 treatment to human islet cells suppressed the expression of inflammatory genes and protected β cells from cytokine-induced apoptosis (110, 111). In addition, GLP-1 RA reduced inflammatory macrophage activation and inflammatory cytokines such as IL-1β and IL-6 in T2D patients (112).

DPP-4is are anti-hyperglycemic drugs that increase insulin secretion and reduce glucose level by preventing the activity of DPP-4 degrading incretins such as GLP-1. These drugs also have anti-inflammatory potential. DPP-4i including sitagliptin suppresses nod-like receptor family, pyrin domain containing 3 (NLRP3) inflammasome in human macrophages *via* inhibition PKC pathway and reduced inflammatory cytokines including TNF-α, IL-6, IL-1β in WAT (113, 114). In human studies, sitagliptin treatment reduced levels of Hb1Ac and inflammatory cytokines in T2D patients (115).

### IL-1 Receptor Antagonist

Hyperglycemia increases the production of IL-1 which drives dysfunction of β cells. Therefore, blocking IL-1 signaling pathway can reduce islet inflammation and delay T2D development. In rodent studies, IL-1 receptor antagonist (IL-1Ra) reduced hyperglycemia and enhanced function of β cells by reducing expression of TNF-α, MCP-1 and IL-6 in liver and islet (47, 48). In a clinical trial, administration of anakinra, IL-1R blockade, increased insulin secretion and improved glycemia in T2D patients versus placebo (116). Canakinumab, an anti-IL-1β antibody, also reduced plasma IL-6 level, leading to improvement of glycemia in T2D patients (117) (**Table 2**).

**Table 2:** Summary of major clinical trials for anti-inflammatory therapies on T2D.

Agent	Mechanism of Action	Phase, ID	Main Findings	Reference
Liraglutide	GLP-1 receptor agonist	Phase III, NCT01620489	Reduction of Hb1Ac and body weight	(118)
Exendin-4	GLP-1 receptor agonist	Phase III, NCT00637273	Reduction of Hb1Ac	(119)
		Phase III, NCT01554618	Ongoing	(120)
Anakinra	IL-1 receptor antagonist	Phase II, NCT00303394	Reduction of glycated hemoglobin level and enhanced secretion of C peptide	(116)
		Phase IV, NCT02236481	Reduction of Hb1Ac	(121)
		Phase IV, NCT00711503	No effects on C peptide level and Hb1Ac levels	(122)
		Phase II, NCT04227769	Ongoing	(123)
Canakinumab	Anti-IL-1β mAb	Phase II, NCT00947427	No effects on C peptide level and Hb1Ac levels	(122)

GLP-1, Glucagon-like peptide-1; Hb1Ac, glycated hemoglobin; mAb, monoclonal antibody.

## Gut Microbiota as Novel Therapeutic Approach for Diabetes

Recently, several studies have demonstrated that gut microbiota has important roles in the development of diabetes. Therefore, targeting gut microbiota has been considered as therapeutic approaches for diabetes.

### Probiotics

Probiotics are live microorganisms regarded as beneficial to host gut microbiota homeostasis. Several studies demonstrated that adequate consumption of probiotics can improve immune responses, insulin resistance, and insulin secretion by modulation of gut microbiota (124–126). For example, *Lactobacillus johnsonii* N62 support maintain IEC barrier by modifying intestinal microbiota and *Lactobacillus rhamnosus* GG is known to attenuate LPS-induced inflammation by reducing TRL4 expression (127–129). *Lactobacillus reuteri* also has been reported that enhance intestinal barrier function and have anti-inflammatory effects by suppressing T cell response or inducing Treg (130). In NOD mouse model, administration of a probiotic combination including *Lactobacillus acidophilus* and *Bifidobacterium lactis* inhibited T1D development by reducing gut permeability, CD8^+^ T cells polarization and increasing Treg population (131). In humans, numerous clinical studies using probiotics for T1D patients are ongoing. Probiotics has been also considered for the prevention of T2D. Treatment of 14 probiotics decreased Hb1Ac level and increased C peptide level in db/db mice by increasing SCFA-producing bacteria and reducing levels of *Escherichia* and inflammatory cytokines (132). This compositional change of gut microbiota contributes to improving intestinal permeability and β cell function by increasing GLP-1 secretion. In clinical studies, administration of *Lactobacillus acidophilus* or *Bifidobacterium bifidum* also enhanced glycemic control and reduced inflammatory cytokines and oxidative stress in T2D patients *versus* placebo (133, 134).

### Fecal Microbiota Transplantation

Recently, fecal microbiota transplantation (FMT) has considered as a therapeutic strategy for diabetes. Oral transfer of fecal bacteria from NOD.Myd88^−/−^ altered gut microbial composition of diabetes-prone NOD, leading to a delay in the progression of T1D (135). In a randomized controlled trial, the transfer of fecal bacteria from healthy donors improved β cell function in T1D patients by reducing CXCR3^+^ T cells (136). Furthermore, a FMT treatment to HFD and streptozotocin-induced T2D mice model improved insulin resistance and destruction of β cells by a decrease of inflammatory cytokines (137). In humans, FMT from lean donors to obese recipients for 6 weeks increased levels of butyrate-producing intestinal microbiota to improve insulin sensitivity (138). This result implies that FMT treatment also promising therapeutic strategy for the prevention of T2D (**Table 3**).

**Table 3:** Summary of current clinical trials evaluating the efficacy of probiotics or FMT on diabetes.

Agent/Procedure	Condition	Phase, ID	Main Findings	Reference
**probiotics**				
*Lactobacillus johnsonii* N6.2	T1D	Phase I/II, NCT02349360	Feasibility of alleviating occurrence of T1D	(139)
*Lactobacillus johnsonii* Probiotic	T1D	Phase II, NCT03961347	Ongoing	(140)
*Lactobacillus rhamnosus* GG and *Bifidobacterium lactis* BB12	T1D	Phase IV, NCT03032354	No effects on maintaining β cell function	(141)
Probiotics (Visbiome)	T1D	Phase II, NCT04141761	Ongoing	(142)
Probiotics (VSL#3)	T1D	Not Applicable, NCT03423589	N/A but completed	(143)
*Lactobacillus Reuteri* DSM 17938	T2D	Not Applicable, NCT01836796	No effects on Hb1Ac levels, but high-dose of Lactobacillus Reuter increased insulin sensitivity and 2nd BA	(144)
Alive multi-strain probiotic mixture	T2D	Not Applicable, NCT03434860	Reduction of HOMA-IR weight, and inflammatory cytokines	(145)
*Lactobacillus acidophilus* NCFM (DB15823)	T2D	Not Applicable, NCT00413348	Unknown	(146)
**Fecal Microbiota Transplantation (FMT)**				
FMT through mid-gut	T2D	Phase II/III, NCT01790711	Unknown	(147)
FMT	T2D and obesity	Not Applicable, NCT03127696	Increased the engraftment of lean-associated microbiota and increased SCFA-producing bacteria	(148)
FMT	T2D and obesity	Phase II, NCT02346669	Unknown	(149)

HOMA-IR, homeostasis model assessment for insulin resistance; BA, bile acid; SCFA, short chain fatty acid.

## Conclusions

Diabetes is a multifactorial disease caused by genetic factors as well as environmental factors that affect abnormal immune modulation, leading to the dysfunction and destruction of pancreatic β cells. Although the mechanisms stimulating the change of the immune responses are diverse, and are not fully understood yet, targeting effector CD4^+^ T cells and CD8^+^ T cells, key mediators of β cell damage, has been the focus of immunotherapeutic approaches to prevent diabetes. Indeed, anti-CD3 mAb therapy such as teplizumab showed promising results including the preservation of β cell function in clinical studies. However, most of initial phase III trials using anti-CD3 mAbs in patients with T1D were failed because they did not meet their primary endpoint. Moreover, several adverse effects were revealed including rash or Epstein-Barr virus (EBV) reactivation. Therefore, novel therapeutic strategies are required to prevent adverse effects for the treatment of T1D.

The aberrant immune modulation *via* obesity is also crucial for the development of T2D. Although the immunomodulatory mechanisms of T2D progression remain unclear, it has been believed that obesity-mediated metabolic stresses such as ER-stress and oxidative stress contributed to T2D development by induction of pro-inflammatory responses. Anti-inflammatory agents such as metformin as well as GLP-1 RA and DPP-4i are widely used to treat T2D patients. Blocking IL-1 signaling is also one of the immunotherapeutic strategies for the treatment of T2D.

Currently, the compositional change of gut microbiota has been associated with T1D and T2D. Although the roles of gut microbiota are still not fully understood, several studies have demonstrated that the presence or absence of specific microbiota can contribute to immune modulation, leading to the development of diabetes by abnormally regulating host metabolisms. Interestingly, FMT has attracted attention as a therapeutic strategy for diabetes. Several studies have reported that FMT can improve β cell destruction and delay the progression of T1D in mouse models or humans. Altogether, numerous studies by immune modulation are ongoing for the prevention and treatment for diabetes patients. And novel therapeutic strategies to minimize adverse effects are still required for the development of immune modulator to prevent T1D and T2D.

## Author Contributions

SF drafted and revised the manuscript. SJ wrote and revised the manuscript. All authors contributed to the article and approved the submitted version.

## Funding

This work was supported by Bio and Medical Technology Development Program (NRF-2017M3A9F3046538), National Research Foundation (NRF-2021R1A2C2009749), Ministry of Health and Welfare (HR18C0012) and faculty research grant from the Yonsei University College of Medicine (6-2018-0098) to SF.

## Conflict of Interest

The authors declare that the research was conducted in the absence of any commercial or financial relationships that could be construed as a potential conflict of interest.

## Publisher’s Note

All claims expressed in this article are solely those of the authors and do not necessarily represent those of their affiliated organizations, or those of the publisher, the editors and the reviewers. Any product that may be evaluated in this article, or claim that may be made by its manufacturer, is not guaranteed or endorsed by the publisher.
